# Tofacitinib Ameliorates Lipopolysaccharide-Induced Acute Kidney Injury by Blocking the JAK-STAT1/STAT3 Signaling Pathway

**DOI:** 10.1155/2021/8877056

**Published:** 2021-01-13

**Authors:** Yang Yun, Jingyu Chen, Xuejiao Wang, Yingzhuo Li, Zhifan Hu, Pingting Yang, Ling Qin

**Affiliations:** ^1^Department of Physiology, China Medical University, Liaoning 110122, China; ^2^Department of Rheumatology and Immunology, First Affiliated Hospital, China Medical University, Liaoning 110001, China

## Abstract

Septic acute kidney injury (AKI) is the most common AKI syndrome in the intensive care unit (ICU), and it accounts for approximately half of AKI cases. Tofacitinib (TOFA) is a pan-Janus kinase (JAK) inhibitor that exhibits potent anti-inflammatory activity in rheumatoid arthritis. However, no study has examined the functional role of TOFA in septic AKI. In the present study, we investigated the protective effects of TOFA on septic AKI and the underlying mechanisms. A lipopolysaccharide- (LPS-) induced AKI model was established in C57BL/6 mice via an intraperitoneal injection of LPS (10 mg/kg). One hour after LPS challenge, the mice were orally administered TOFA (5, 10, or 15 mg/kg) every 6 h until sacrifice at 24 h. We found that TOFA significantly ameliorated LPS-induced renal histopathological changes and dysfunction. TOFA also suppressed the expression levels of proinflammatory cytokines (TNF-*α*, IL-1*β*, IL-6, and IFN-*γ*) and the parameters of oxidative stress (MDA, GSH, SOD, and CAT) in kidney tissues. These results may be associated with the inhibitory effect of TOFA on the JAK-STAT1/STAT3 pathway, which was significantly activated by LPS challenge. TOFA treatment also inhibited LPS-induced activation of the TLR4/NF-*κ*B pathway. In conclusion, we revealed that TOFA had a protective effect on LPS-induced AKI, and it may be a promising therapeutic agent for septic AKI.

## 1. Introduction

Acute kidney injury (AKI) is a common clinical syndrome that is characterized by severe tubular cell injury and rapid renal dysfunction [1]. Survivors also have an increased risk of progression to chronic kidney disease [2]. Sepsis is the major trigger of AKI in critically ill patients, and it contributes up to 50% of mortality in intensive care unit (ICU) patients [3]. Therefore, it is necessary to reveal the pathophysiological mechanism of AKI in sepsis and find effective treatments to prevent this devastating disease.

A growing body of evidence indicates that the inflammatory response inherent to sepsis-related endotoxemia is a direct mechanism of AKI [4]. Endotoxemia primarily results from lipopolysaccharide (LPS), which is the major component of endotoxin released from the cell wall of Gram-negative bacteria [5]. During endotoxemia, LPS-induced Toll-like receptor 4 (TLR4) activation promotes the expression of proinflammatory cytokines, including tumor necrosis factor-*α* (TNF-*α*), interleukins (IL-1*β* and IL-6), and interferon (IFN), via the transcription of nuclear factor-*κ*B (NF-*κ*B), which is a crucial regulator of the immune system [6–8]. From this view, blockade of cytokine pathways may be a potential approach to prevent the development of septic AKI.

The Janus kinase- (JAK-) signal transducer and activator of transcription (STAT) pathway is the major signaling cascade downstream of type I and type II cytokine receptors. JAKs are effective therapeutic targets for various cytokine-driven autoimmune and inflammatory diseases [9, 10]. Tofacitinib (TOFA) is a first-generation JAK inhibitor that blocks the signaling cascades of the c-chain-using cytokines, such as IL-6, which interacts with JAK1 and JAK3. TOFA also blocks Gp130-using cytokines and signaling downstream of IFNs. Therefore, TOFA may have an inhibitory effect on the proinflammatory cytokines involved in septic AKI. Although TOFA has been shown to be effective in the treatment of rheumatoid arthritis [11], psoriasis arthritis [12], ulcerative colitis [13], and alopecia areata [14], its role in septic AKI remains unknown.

LPS-induced AKI in mice recapitulates several of the clinical manifestations observed in human septic AKI. Therefore, it is one of the most commonly used animal models to investigate the pathogenesis and potential treatment of septic AKI [15, 16]. The present study used this animal model to examine the effects of TOFA on septic AKI. We found that pharmacological inhibition of the JAK-STAT pathway significantly ameliorated the clinical phenotype of AKI, reversed oxidative stress, and downregulated the expression of proinflammatory cytokines and the subsequent activation of downstream pathways. Our results suggest a potential role of TOFA in the treatment of septic AKI.

## 2. Materials and Methods

### 2.1. Reagents

LPS (Sigma-Aldrich, St. Louis, MO, USA) was dissolved in 0.9% saline and administered intraperitoneally at a dose of 10 mg/kg, as reported previously [17]. TOFA citrate (Pfizer, Freiburg, GER) was dissolved in 0.5% methyl cellulose/0.25% Tween-20 in PBS and was orally administered via gavage every 6 h. The doses of TOFA used in this study were based on a previous study [18].

### 2.2. Animals

Specific pathogen-free wild-type (C57BL/6) mice were purchased from the Vital River Laboratory (BJ, CHN) at the age of 4 weeks. Male mice (6-8 weeks old) were selected for the experiment. All animals were reared in standard animal cages under environmentally controlled laboratory conditions (12 h/12 h light/dark cycle, 22 ± 2°C, 40-80% humidity) with ad libitum access to food and water. All efforts were made to minimize animal suffering. The animals were maintained and treated in compliance with the policies and procedures detailed in the “Guide for the Care and Use of Laboratory Animals” of the National Institutes of Health. The Animal Care and Use Committee of China Medical University reviewed and approved the animal experimental protocols of the “Guide” and the treatment procedures (No. KT2018060).

### 2.3. Experimental Procedure

**Figure 1:**
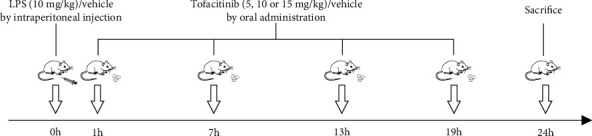
Experimental schedule. Mice were administered LPS (10 mg/kg) or vehicle via intraperitoneal injection. One hour later, TOFA (5, 10, or 15 mg/kg) or vehicle was given via gavage every 6 h until sacrifice at 24 h.

As shown in Figure 1, 50 mice were randomly allocated into the following five groups (*n* = 10): control, LPS, LPS+TOFA (5 mg/kg), LPS+TOFA (10 mg/kg), and LPS+TOFA (15 mg/kg) groups. LPS (10 mg/kg) or vehicle was administered intraperitoneally. One hour after LPS or vehicle challenge, TOFA (5, 10, or 15 mg/kg) or vehicle was orally administered via gavage every 6 h. Blood and kidney tissue samples were collected 24 h after LPS treatment.

### 2.4. Renal Function Assessment

Serum was separated via centrifugation at 5,000 rpm for 15 min at 4°C. The levels of mouse blood urea nitrogen (BUN) and serum creatinine (Scr) were detected using a BUN assay kit (C013-2-1, Jiancheng, NJ, CHN) and a Scr assay kit (C011-2-1, Jiancheng, NJ, CHN), respectively. Experimental procedures were strictly followed according to the manufacturer's protocols. Each sample was tested at least three times, and the average value was taken.

### 2.5. Renal Histological Analysis

Mice were anesthetized and transcardially perfused with 0.1 M PBS (pH 7.5, 4°C) 24 h after LPS treatment. Unilateral kidney samples were harvested from mice and post-fixed in a solution containing 4% paraformaldehyde in PBS overnight at 4°C. The kidney tissue was embedded in paraffin after dehydration and sliced into 5 *μ*m sections. The sections were stained with hematoxylin and eosin (H&E) to observe renal damage using light microscopy (×200, BX53, Olympus, TKY, JP). The percentage of tubules that displayed cellular necrosis, loss of brush border, cast formation, vacuolization, and tubular dilation in 10 different fields was scored as follows: 0 = none, 1 ≤ 10%, 2 = 11–25%, 3 = 26–45%, 4 = 46–75%, and 5 ≥ 76% [19].

### 2.6. Quantitative Real-Time Polymerase Chain Reaction (qRT-PCR)

Expression of proinflammatory cytokines TNF-*α*, IL-1*β*, IL-6, and IFN-*γ* in kidney tissue was detected using qRT-PCR. Briefly, total RNA was extracted from homogenized kidney tissue using a TRI pure Reagent assay kit (RP1001, BioTeke, BJ, CHN) according to the manufacturer's instructions. The RNAs from each group were reverse-transcribed using a reverse transcription kit (PR6502, BioTeke, BJ, CHN) to obtain the corresponding cDNA. The analysis of gene expression was performed in the Exicycler 96 Real Time PCR System (BIONEER, Daejeon, ROK) using a SYBR Green kit (SY1020, Solarbio, BJ, CHN). The changes in mRNA were normalized to the control (*β*-actin). Each sample was tested at least three times, and the average value was taken. The sequences of primers used for qRT-PCR are listed in Table 1.

### 2.7. Western Blot Analysis

Total proteins of unilateral kidney samples were lysed in RIPA lysis buffer and extracted with a T-PER protein extract kit (WLA019, Wanleibio, SY, CHN) according to the manufacturer's instructions. The protein concentrations were measured with a bicinchoninic acid (BCA) assay kit (WLA004, Wanleibio, SY, CHN). Equal amounts of proteins were separated in 10% sodium dodecyl sulfate-polyacrylamide gels (SDS-PAGE) and transferred onto polyvinylidene fluoride (PVDF) membranes (IPVH00010, Millipore, MA, USA). The membrane was blocked with 5% nonfat milk for one hour at room temperature. The membrane was incubated with rabbit anti-mouse primary antibodies that recognized TNF-*α* (WL01581, 1 : 500, Wanleibio, SY, CHN), IL-1*β* (WL00891, 1 : 500, Wanleibio, SY, CHN), IL-6 (WL02841, 1 : 1000, Wanleibio, SY, CHN), IFN-*γ* (WL02440, 1 : 500, Wanleibio, SY, CHN), TLR4 (WL00196, 1 : 1000, Wanleibio, SY, CHN), p65 (WL01980, 1 : 500, Wanleibio, SY, CHN), p-p65 (WL02169, 1 : 500, Wanleibio, SY, CHN), STAT1 (14994 T, 1 : 1000, CST, CA, USA), STAT3 (4904 T, 1 : 2000, CST, CA, USA), p-STAT1 (ab109461, 1 : 1000, Abcam, Cambs, UK), p-STAT3 (9145 T, 1 : 2000, CST, CA, USA), and *β*-actin (4970 T, 1 : 1000, CST, CA, USA) at 4°C overnight. Membranes were washed three times and incubated with a goat anti-rabbit secondary antibody (A0208, 1 : 5000, Beyotime, SH, CHN) for two hours at room temperature in the dark. Band intensities were quantified using the ImageJ software. Each sample was tested at least three times, and the average value was taken.

### 2.8. Oxidative Stress Parameter Measurements

Kidney tissue blocks were homogenized at a *w*/*v* ratio of 10% in 0.9% cold saline and centrifuged at 2,500 rpm for 10 minutes at 4°C. The supernatant was collected for the detection of oxidative stress markers. The total protein concentration of kidney homogenates was measured using a bicinchoninic acid (BCA) assay kit (WLA004, Wanleibio, SY, CHN). The levels of malondialdehyde (MDA) and reduced glutathione (GSH) and the activities of superoxide dismutase (SOD) and catalase (CAT) were measured using corresponding assay kits (Jiancheng, NJ, CHN) according to the manufacturer's instructions. Each sample was tested at least three times, and the average value was taken.

### 2.9. Statistical Analysis

Statistical analyses were performed using the SPSS 18.0 software (SPSS Inc., Chicago, IL, USA). All data are expressed as the means ± standard error of the mean (SEM). Comparisons of three or more groups were performed using one-way analysis of variance (ANOVA) followed by Tukey's post hoc test. Statistical significance was accepted at *P* < 0.05.

## 3. Results

### 3.1. TOFA Treatment Ameliorated Renal Dysfunction

**Figure 2:**
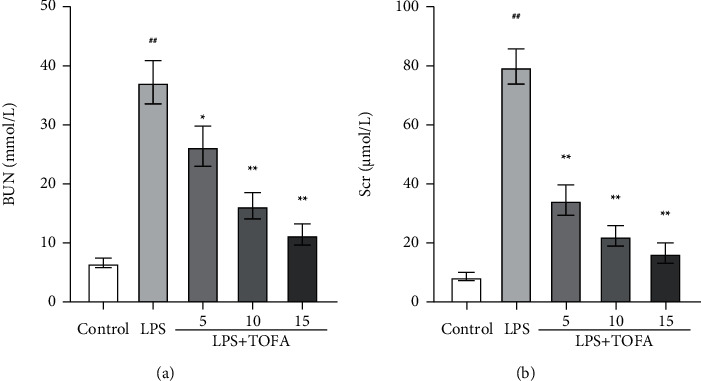
Effects of TOFA on renal dysfunction in LPS-induced AKI mice. BUN (a) and Scr (b) were used to assess renal function. Values represent the means ± SEM (*n* = 10 in each group). One-way ANOVA followed by Tukey's post hoc test: ^##^*P* < 0.01 compared to the control group, ^∗^*P* < 0.05 compared to the LPS group, ^∗∗^*P* < 0.01 compared to the LPS group.

As important indexes of renal injury, BUN and Scr were detected to assess renal function. The levels of BUN (a) and Scr (b) in the LPS group were obviously elevated at 24 h compared to the control group (Figure 2, ANOVA followed by Tukey's post hoc test). The levels of BUN and Scr were dose-dependently restored in the LPS+TOFA groups (Figure 2, ANOVA followed by Tukey's post hoc test), which indicated that TOFA exerted a protective effect on renal function in LPS-induced AKI.

### 3.2. TOFA Treatment Attenuated Renal Histological Injury

**Figure 3:**
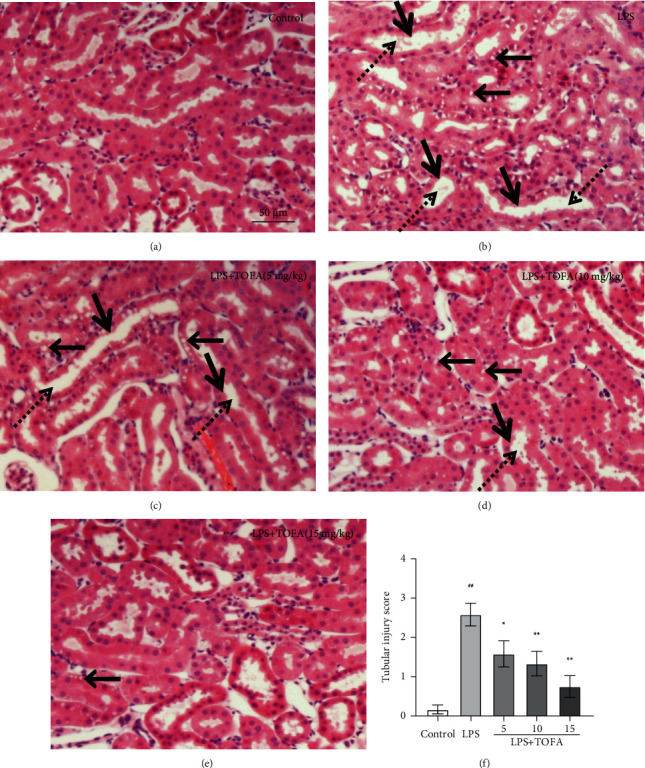
Effects of TOFA on renal histopathological changes in LPS-induced AKI mice. (a) Control group. (b) LPS group. (c) LPS+TOFA (5 mg/kg) group. (d) LPS+TOFA (10 mg/kg) group. (e) LPS+TOFA (15 mg/kg) group. (f) Tubular injury score (H&E staining, magnification ×200). The arrow point, thick arrow point, and arrow point with a dotted line represent tubular vacuolization, loss of brush border, and tubular dilation, respectively. Scale bar indicates 50 *μ*m. One-way ANOVA followed by Tukey's post hoc test: ^##^*P* < 0.01 compared to the control group, ^∗^*P* < 0.05 compared to the LPS group, ^∗∗^*P* < 0.01 compared to the LPS group.

To investigate the protective effect of TOFA on LPS-induced AKI, renal histological changes were analyzed using H&E staining. The LPS group showed obvious renal histopathological changes, including tubular vacuolization, loss of brush border, and tubular dilation compared to the control group (Figures 3(a) and 3(b)). These pathological changes were dose-dependently attenuated in the LPS+TOFA groups (Figures 3(c)–3(e)). We used the tubular injury score to quantitatively evaluate the histopathological changes and confirmed that TOFA efficiently prevented LPS-induced renal injuries (Figure 3(f), ANOVA followed by Tukey's post hoc test).

### 3.3. TOFA Treatment Decreased Renal Proinflammatory Cytokine Production

**Figure 4:**
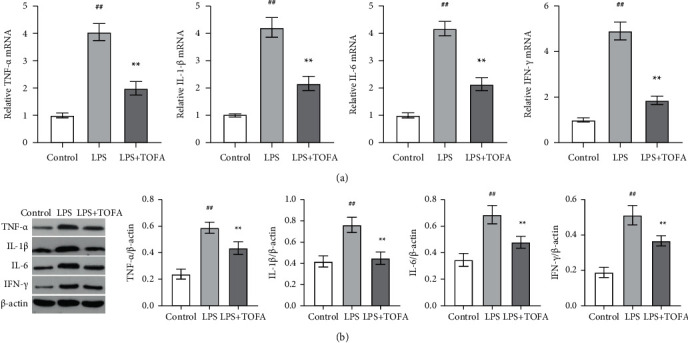
Effects of TOFA on renal proinflammatory cytokine production in LPS-induced AKI mice. qRT-PCR (a) and Western blot (b) were used to measure renal mRNA expression and protein levels of TNF-*α*, IL-1*β*, IL-6, and IFN-*γ* in the control, LPS, and LPS+TOFA (10 mg/kg) groups. Values represent the means ± SEM (*n* = 10 in each group). One-way ANOVA followed by Tukey's post hoc test: ^##^*P* < 0.01 compared to the control group, ^∗^*P* < 0.05 compared to the LPS group, ^∗∗^*P* < 0.01 compared to the LPS group.

To assess the anti-inflammatory effects of TOFA, we selected samples from the control, LPS, and LPS+TOFA (10 mg/kg) groups to measure the levels of proinflammatory cytokines in kidney tissue using qRT-PCR and Western blot. As shown in Figure 4, the mRNA expression (a) and protein levels (b) of TNF-*α*, IL-1*β*, IL-6, and IFN-*γ* in the LPS group were significantly elevated compared to the control group (ANOVA followed by Tukey's post hoc test). TOFA dramatically rescued the increased expression of TNF-*α*, IL-1*β*, IL-6, and IFN-*γ* (Figure 4, ANOVA followed by Tukey's post hoc test), which suggests that TOFA inhibited excessive renal proinflammatory cytokine production in LPS-induced AKI.

### 3.4. TOFA Treatment Reduced Oxidative Stress

**Figure 5:**
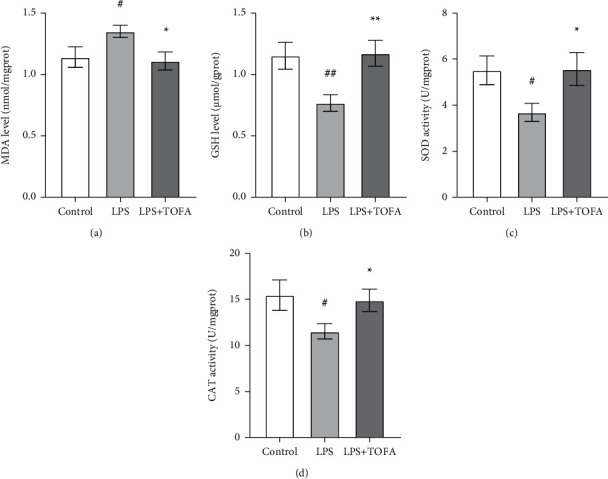
Effects of TOFA on renal oxidative stress in LPS-induced AKI mice. MDA (a), GSH (b), SOD (c), and CAT (d) were used to assess renal oxidative stress. Values represent the means ± SEM (*n* = 10 in each group). One-way ANOVA followed by Tukey's post hoc test: ^#^*P* < 0.05 compared to the control group, ^##^*P* < 0.01 compared to the control group, ^∗^*P* < 0.05 compared to the LPS group, ^∗∗^*P* < 0.01 compared to the LPS group.

Previous research found that oxidative stress participated in the development and progression of LPS-induced AKI [20, 21]. Therefore, we tested whether TOFA administration reduced oxidative stress in LPS-induced AKI. As shown in Figure 5, LPS injection pronouncedly depleted renal levels of antioxidants (SOD, CAT, and GSH) and markedly increased renal levels of the lipid peroxidation product MDA compared to the control group. TOFA administration significantly restored the changes in oxidative stress parameters (Figure 5).

### 3.5. TOFA Treatment Restrained the Activation of the JAK-STAT1/STAT3 and TLR4/NF-*κ*B Signaling Pathways

**Figure 6:**
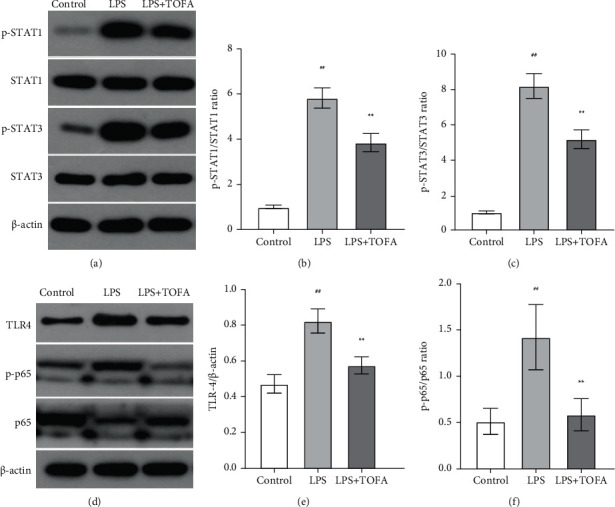
Effects of TOFA on the JAK-STAT and TLR4/NF-*κ*B signaling pathways in LPS-induced AKI mice. Western blot was used to analyze the expression of STAT1 (a), p-STAT1 (a), STAT3 (a), p-STAT3 (a), TLR4 (d, e), p65 (d), and p-p65 (d) and the phosphorylation ratios of STAT1 (b), STAT3 (c), and p65 (f) in the control, LPS, and LPS+TOFA (10 mg/kg) groups. Values represent the means ± SEM (*n* = 10 in each group). One-way ANOVA followed by Tukey's post hoc test: ^##^*P* < 0.01 compared to the control group, ^∗∗^*P* < 0.01 compared to the LPS group.

We further used Western blot analyses to investigate the effects of TOFA on the JAK-STAT signaling pathway in LPS-induced AKI mice. As illustrated in Figures 6(b) and 6(c), the phosphorylation ratios of STAT1 and STAT3 were significantly facilitated in the LPS group compared to the control group (ANOVA followed by Tukey's post hoc test). TOFA administration (10 mg/kg) reduced the LPS-induced increase in STAT1 and STAT3 phosphorylation (Figures 6(b) and 6(c)).

We also examined whether TOFA administration affected the TLR4/NF-*κ*B signaling pathway, which has been shown to be activated by LPS previously [8]. As shown in Figures 6(d) and 6(e), the level of TLR4 in the LPS group was significantly elevated compared to the control group, and TOFA inhibited the increase in TLR4 expression (ANOVA followed by Tukey's post hoc test). Western blot analysis of p65 phosphorylation showed a similar tendency.

## 4. Discussion

Many efforts have been made to improve the treatment for AKI over the last several decades, but current therapies are still unable to significantly reduce mortality. The manipulation of cytokine-mediated inflammation is a new approach to prevent and/or treat septic AKI. In this study, we showed that the oral application of a drug that preferentially targets JAK1 and JAK3, TOFA, had beneficial effects in LPS-induced AKI mice as indicated by (a) a significant amelioration of BUN and Scr elevation, renal histological changes, and oxidative stress dysregulation, and (b) suppression of the production of proinflammatory cytokines and the activation of downstream signaling pathways. Our results highlight the potential application of TOFA for the treatment of septic AKI.

The renal cortex, which includes renal tubules and renal corpuscles, is the outer portion of the kidney where ultrafiltration occurs. The proximal tubule is the portion of the duct system of the nephron in the kidney. Consistent with previous reports [21], our histological results showed that LPS-induced AKI was primarily related to renal tubular injuries, accompanied by the elevation of BUN and Scr levels and the dysregulation of oxidative stress markers. We also found that LPS challenge substantially increased the expression of proinflammatory cytokines (TNF-*α*, IL-1*β*, IL-6, and IFN-*γ*) in mouse renal tissues. Numerous studies demonstrated that the excessive release of proinflammatory cytokines, such as IL-1*β*, IL-6, and TNF-*α*, triggered the pathophysiological abnormities of sepsis [22–24] and played prominent roles in septic AKI [25, 26]. Our study confirmed these previous findings and further revealed that LPS activated the STAT pathway in renal tissues. The STAT pathway and its upstream kinases play a role in initiating the inflammatory response to LPS in the lung, liver, hypothalamus, and joints [27–29]. Some evidence also showed STAT activation in the renal tubular cells of ischemia/reperfusion injury kidney [30–33]. However, the mechanism of LPS-induced STAT activation in the kidney remained unclear, but it likely involves a complex interplay between inflammatory mediators and renal cells. It has been known that LPS can be filtered through Bowman's capsule and into the tubular fluid. Once in the tubular space, LPS directly interacts with tubular epithelial cells, which recognize it via a TLR4-dependent mechanism and, then, activates the NF-*κ*B pathway to promote the production and release of proinflammatory cytokines [34]. IL-6 and IFN-*γ* are two possible candidates for the activation of STATs. IL-6-induced STAT3 activation was observed in mice with HgCl_2_-induced AKI [35], and IFN-*γ* has been shown to activate STAT1 in glomerular mesangial cells in an aristolochic acid nephropathy mouse model [36]. Based on these evidences, we speculated that LPS likely did not directly activate STATs in renal cells and STAT activation may occur indirectly via IL-6 and IFN-*γ*, which are generated downstream of LPS. This idea is supported by our current findings that LPS induced an increase in TLR4 expression and NF-*κ*B activation. However, this possibility needs to be further examined in the future work using IL-6- or IFN-*γ*-deficient mice.

The JAK-STAT pathway transmits signals from extracellular ligands, including many cytokines and chemokines, directly to the nucleus to induce a variety of cellular responses [37]. Based on the known role of JAK-STAT activation in response to cytokines and chemokines, it is not surprising that JAK-STAT activation is involved in the pathogenesis of AKI. Therefore, we used TOFA to treat LPS-induced AKI. Our work confirmed that TOFA inhibited STAT1 and STAT3 activation in LPS-challenged kidneys, which consequently reduced tubular injury scores, oxidative stress markers, and BUN and Scr levels. We also found that TOFA suppressed LPS-induced cytokine overexpression in kidney tissues. This result is understandable, because STATs are important transcription factors that regulate the expression of inflammatory genes, including cytokines [38]. In line with this, other previous studies also showed that the inhibition of STAT phosphorylation (p-STAT) significantly downregulates the production of proinflammatory cytokines during the process of renal inflammation and injury [39]. We also found that TOFA inhibited activation of the TLR4/NF-*κ*B pathway. This may be attributable to the complicated cross-talk between different signaling pathways. Taken together, these results suggest that the JAK-STAT pathway is a key route in the signaling cascade of cytokine-mediated AKI, and JAK inhibitors may prevent and/or treat AKI via blockade of the feedback loop of proinflammatory cytokines. Notably, a protective or reparative role of STAT6 was also observed in the kidney following ischemia-reperfusion injury [40] or diphtheria toxin-induced AKI [41]. Therefore, the practical effect of TOFA may depend on the pathogen and progression of AKI. Various JAK isoform-selective inhibitors are being developed and tested in preclinical models and early-stage clinical trials [42]. Future studies should assess the effects of these reagents on sepsis-associated AKI to better define the molecular events underlying the efficacy of TOFA.

## 5. Conclusions

In summary, the present study demonstrated for the first time that TOFA played a protective role in LPS-induced AKI by inhibiting the JAK-STAT signaling pathway. These data suggest that in addition to improving autoimmune diseases and preventing transplant rejection, TOFA may be a potential candidate for the treatment of septic AKI in the clinic.

## Figures and Tables

**Table 1:** Primers of qRT-PCR analysis (5′⟶3′).

Gene	Forward primer	Reverse primer
TNF-*α*	5′-CCAGACCCTCACACTCACAAA-3′	5′-GGCTGACGGTGTGGGTGAG-3′
IL-1*β*	5′-TCCTGTGTAATGAAAGACGGC-3′	5′-TGCTTGTGAGGTGCTGATGTA-3′
IL-6	5′-ATGGCAATTCTGATTGTATG-3′	5′-GACTCTGGCTTTGTCTTTCT-3′
IFN-*γ*	5′-AGCAACAACATAAGCGTCAT-3′	5′-CCTCAAACTTGGCAATACTCA-3′
*β*-actin	5′-CTGTGCCCATCTACGAGGGCTAT-3′	5′-TTTGATGTCACGCACGATTTCC-3′

## Data Availability

The data used to support the findings of this study are included within the article.
